# HCV Detection, Discrimination, and Genotyping Technologies

**DOI:** 10.3390/s18103423

**Published:** 2018-10-12

**Authors:** Shrikant Dashrath Warkad, Satish Balasaheb Nimse, Keum-Soo Song, Taisun Kim

**Affiliations:** Institute for Applied Chemistry and Department of Chemistry, Hallym University, Chuncheon 200-702, Korea; shrikant.warkad@hallym.ac.kr (S.D.W.); satish_nimse@hallym.ac.kr (S.B.N.); hanlimsk@empal.com (K.-S.S.)

**Keywords:** HCV, detection, genotyping, quantification, viral load, RT-PCR, nucleic acids, viruses

## Abstract

According to the World Health Organization (WHO), 71 million people were living with Hepatitis C virus (HCV) infection worldwide in 2015. Each year, about 399,000 HCV-infected people succumb to cirrhosis, hepatocellular carcinoma, and liver failure. Therefore, screening of HCV infection with simple, rapid, but highly sensitive and specific methods can help to curb the global burden on HCV healthcare. Apart from the determination of viral load/viral clearance, the identification of specific HCV genotype is also critical for successful treatment of hepatitis C. This critical review focuses on the technologies used for the detection, discrimination, and genotyping of HCV in clinical samples. This article also focuses on advantages and disadvantages of the reported methods used for HCV detection, quantification, and genotyping.

## 1. Introduction

Hepatitis C virus (HCV) causes Hepatitis C, a liver disease [1]. The Global Hepatitis report 2017 by the World Health Organization (WHO) indicates that approximately 71 million people were living with Hepatitis C virus infection worldwide in 2015 [2]. Chronic HCV infection often advances into cirrhosis or liver cancer [3,4]. Each year, about 399,000 HCV-infected people succumb to cirrhosis, hepatocellular carcinoma, and liver failure [5]. Therefore, screening of HCV infection is mandatory in many epidemiologic settings to begin the appropriate treatment. However, various genotypes and subtypes of HCV pose complications in the treatment resulting in increased mortality attributable to HCV infection [6].

**Figure 1:**
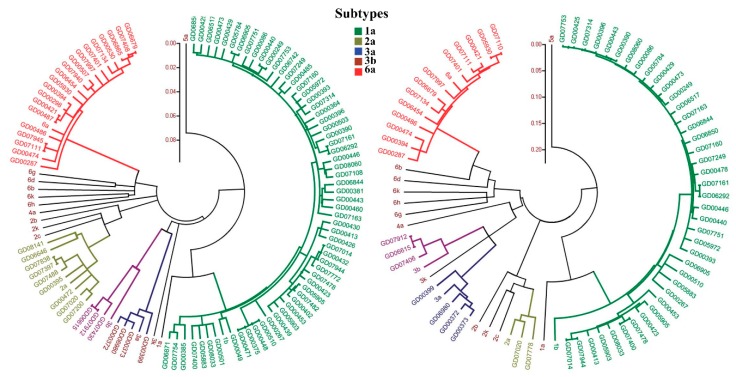
Phylogenetic tree of hepatitis C virus (HCV) sequences. Left, phylogenetic tree constructed based on core sequences. Right, phylogenetic tree constructed based on NS5B sequences. Subtypes 1a, 2a, 3a, 3b, and 6a are shown with 5 different colors in the phylogenetic tree. (Reproduced with permission from [8]).

HCV is an enveloped, single-stranded RNA virus with roughly 9500 nucleotides that codes for a polyprotein consisting of around 3000 amino acids [7]. As shown in Figure 1, the HCV genomic RNA analysis reveals extraordinary heterogeneity in the structural and nonstructural regions of this virus [8]. HCV has 7 genotypes and more than 90 subtypes with diverse patterns of geographic distribution. Worldwide proportions of HCV genotypes 1, 3, 2, 4, 6, and 5 are 46.2%, 30.1%, 9.1%, 8.3%, 5.4%, and 0.8%, respectively [9,10]. The accurate detection of an HCV genotype and subtype is critical for suitable antiviral treatment and cure [11]. According to WHO guidelines 2016, for the screening, care, and treatment of chronic HCV infections, the detection of HCV genotypes is crucial to select the particular combination of drugs [12]. WHO also recommends that a nucleic acid test is vital to decide the treatment for hepatitis C. According to the European Association for the Study of the Liver (EASL) the pangenotypic regimens are available for HCV treatment. However, EASL also recommends the genotype specific regimens for effective treatment of HCV infections [13].

The direct-acting antivirals including sofosbuvir, simeprevir, ledipasvir, ombitasvir, dasabuvir, and other drugs such as pegylated interferon-α, ribavirin are used for the treatment of HCV infection [14]. The duration of treatment involving monotherapy or multi-drug therapy critically depends on HCV genotypes [15]. Therefore, the precise treatment of HCV requires precise detection and discrimination of the HCV genotypes [16].

Currently, there are several methods in use for the detection and genotyping of HCV in clinical samples. The antibody-based assays are sensitive and specific for the detection of HCV infection. However, molecular assays including nucleic acid amplification techniques (NATs) that are qualitative and quantitative for the analysis of HCV RNA are preferred for their HCV genotyping ability required for the definitive therapy [17,18]. Even though NATs typically require a significant investment in equipment, training, and infrastructure, these techniques have become increasingly critical diagnostic tools [19,20].

At present, various NATs including real-time PCR (RT-PCR) [21,22], line-probe assay [23], heteroduplex mobility analysis [24], and restriction fragment length polymorphism [25] are in practice for HCV genotyping in clinical specimens. The high sequence heterogeneity among the types and subtypes of this virus poses a major challenge for HCV genotyping. Hence, sequence analysis remains the gold standard for genotyping of HCV. Moreover, due to the low agreement rate between different methods [26,27] sequence analysis of specific regions of HCV RNA is used as a gold standard to resolve the discrepant results [28]. Even though the sequence analysis is an accurate HCV genotyping method, longer turn around time, cost, and requirement of highly trained professionals are the significant disadvantages of this method in limited resource settings. A rapid, simple, precise, and inexpensive HCV genotyping method is inevitable for the execution of proper treatment regime in the management of hepatitis C. Therefore, there are continuous research and development opportunities in HCV genotyping and diagnosis technologies [29].

**Figure 2:**
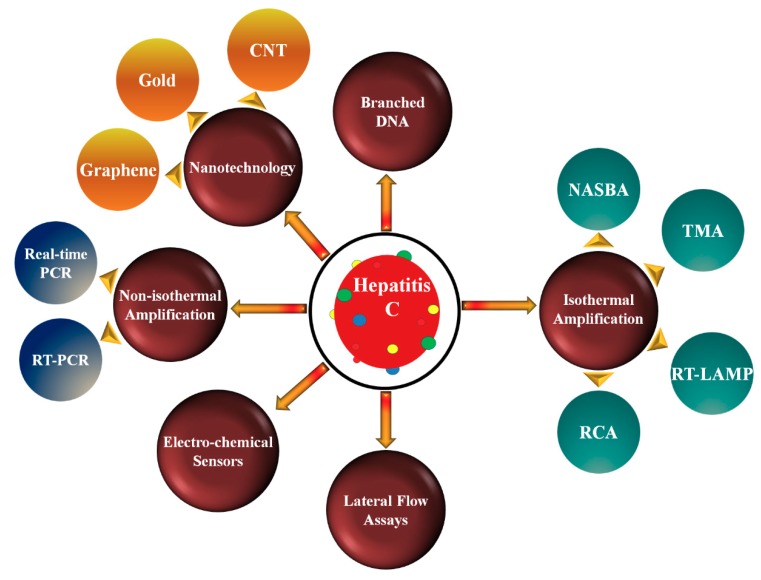
Various technologies used for the qualitative and quantitative detection of HCV. RT-PCR, Reverse transcription polymerase chain reaction; NASBA, Nucleic acid sequence-based amplification; TMA, Transcription-mediated amplification; RT-LAMP, Reverse transcription loop-mediated isothermal amplification; RCA, Rolling circle amplification; CNT, Carbon nano-tube.

In this critical review, we have discussed various technologies used for qualitative and quantitative detection of HCV as shown in Figure 2. The advantages and disadvantages of these methods are also elaborated.

## 2. Nucleic Acid Amplification Technologies

As mentioned earlier, HCV is an RNA virus. Hence, a reverse transcription is always pre-requisite to PCR amplification, which requires a DNA template. Therefore, efficiency of the reverse transcription reaction often dictates the sensitivity of the detection method.

### 2.1. Non-Isothermal Amplification Methods

#### 2.1.1. Reverse Transcription Polymerase Chain Reaction (RT-PCR)

Reverse transcription polymerase chain reaction (RT-PCR) converts RNA molecules into their complementary DNA (cDNA) sequences by the aid of reverse transcriptase, followed by amplification of the newly synthesized cDNA in a standard PCR [30]. RT-PCR is used in molecular biology to detect RNA expression and to clone expressed genes [31]. Further, it is also extremely common to use RT-PCR for the conversion of RNA viral genomes to cDNA templates [32]. RT-PCR can be performed on fresh-frozen tissue or body fluids (e.g., plasma, serum, urine) in the two-tube or single-tube experiment. In the two-tube experiment, different enzymes and buffer conditions are required for RT-PCR, whereas for a single-tube experiment the same enzymes and buffer conditions are used for the PCR after reverse transcription [33,34].

Meng et al. reported an RT-PCR assay using two sets of primers/probes and a specific armored RNA as internal control. They reported that their assay showed the limit of detection (LOD) of 38.99 IU/mL with significant specificity and sensitivity in 109 clinical samples [35]. The advantage of this assay was that it could detect all HCV genotypes. However, it failed to genotype individual HCV genotypes.

The AMPLICOR RT-PCR showed a LOD of 50 IU/mL in qualitative test and 600 IU/mL in quantitative test [36]. To study reproducibility and applicability for quantification, Lee et al. developed an RT-PCR assay using two sets of primers/probes and a specific armored RNA as internal control. The quantitative range of this assay was in the linear range of 500 to 500,000 IU/mL. This assay is suitable for determining the viral load and treatment monitoring. The RT-PCR method is a highly sensitive and accurate for the diagnosis of HCV infection, patient response to therapy, and to monitor the efficacy of the drug during therapy [37].

As mentioned earlier, the HCV genotyping is a substantial predictor of the response to antiviral therapy. Therefore, many scientists have developed the HCV genotyping assays. Nakatani et al. reported the use of two triplex reaction sets, one to detect genotypes 1a, 1b, and 3a, and another to detect genotypes 2a, 2b, and 2c. This assay showed the overall sensitivity of 97.0%, detecting 295 of the 304 clinical specimens [38]. The LOD of this assay was reported to be 125 IU/mL for genotype 3a and 250 IU/mL for genotypes 1b and 2b. The LOD for genotype 1a was 500 IU/mL. High LOD of this assay has a disadvantage for its application in screening of samples for HCV infection. The asymptomatic patients and carriers of HCV may have a lower viral load than this assay can detect.

RT-PCR is a sensitive method because it can perform exponential amplification of template RNA. The specificity of RT-PCR is primarily depended on the specificity of the primers for a particular gene in consideration during cDNA synthesis. RT-PCR also has some complexity and problems associated with its reproducibility and specificity. Moreover, when it is used as a quantitative method, it suffers from the problems inherent in traditional PCR [39]. The quantification studies can be technically challenging due to the existence of numerous sources of variation including template concentration and amplification efficiency [40]. The exponential growth of the cDNA during multiple PCR cycles results in inaccurate end-point quantification due to the non-linearity [41].

#### 2.1.2. Real-Time PCR (RT-PCR)

After its invention in 1984 by Kary Mullis, the PCR has found many applications in the qualitative and quantitative analysis of genomic materials. The quantitative polymerase chain reaction (Q-PCR) method is vital for the determination of viral load in clinical samples [42,43,44]. The automated RT-PCR is a widespread technique for the qualitative and quantitative analysis of viral nucleic acids. RT-PCR is profoundly used as a standard method for comparison with the new assays and its ability in the quantification of HCV RNA for therapy monitoring [45].

Vermehren et al. compared two real-time reverse transcriptase PCR-based assays (RealTime HCV and Cobas Ampliprep/Cobas TaqMan HCV [CAP/CTM]) and one signal amplification-based assay (the Versant HCV RNA, version 3.0, branched DNA [bDNA] assay) for their capabilities to measure the viral load of HCV genotypes 1 to 5 in clinical specimens [46]. The LOD of RealTime HCV and CAP/CTM assays were 16.8 and 10.3 IU/mL, respectively. The RT-PCR-based assays showed similar and linear HCV RNA quantification capacities. However, they differed in the sensitive detection of genotypes 1 and 4. Cobb et al. demonstrated the differences between commercially available RT-PCR assays in their review [47]. One of the significant disadvantages of RT-PCR assays is that the result interpretation is very complicated. Laperche et al., in their recent study, reported that the interpretation of RT-PCR results for HCV RNA is not easy for the samples containing viral load below the quantification limits. In their study, they found that 55.6% of the laboratories did not follow the recommendations for the analysis of their results, leading to ambiguous conclusions [48].

Sonia et al. reported an assay based on HCV-RNA detection by SYBR Green RT-PCR [49]. The assay showed an LOD of 5000 copies/mL with an efficiency of 100% for the detection of HCV in dried blood spot specimens. Even though, the assay showed very high sensitivity (99.1%) and specificity (100%), this assay is not practical for the screening of HCV in general population due to its very high detection limit. A significant disadvantage of RT-PCR is that it requires expensive equipment and reagents. For high sensitivity and specificity, sound experimental design and an in-depth understanding of standardization methods are necessary for accurate conclusions [50].

As shown in Table 1, both TaqMan^®^ based assay and SYBR^®^-Green based assay have their own advantages and disadvantages.

### 2.2. Isothermal Amplification Methods (IMA)

The isothermal amplification methods (IMA) are known for their simplicities for the detection of DNA and RNA in the clinical samples. In isothermal amplification methods, a water bath or heat block is used instead of a thermal cycler [51]. The applications isothermal amplification methods have grown significantly in recent years, as they are simple and highly applicable for rapid molecular diagnostics.

Many scientists have tried to miniaturize the isothermal amplification methods to be applicable for rapid diagnosis of pathogens. Rodriguez-Manzano et al. reported a single-molecule counting method, a miniaturized IAM, by using an unmodified cell phone camera [52]. They developed a visual readout system for digital single-molecule amplification of RNA and DNA by using (i) colorimetric amplification-indicator dyes and (ii) optimal ratiometric image-process to accomplish a readout. This method is robust to the lighting conditions and camera hardware.

**Figure 3:**
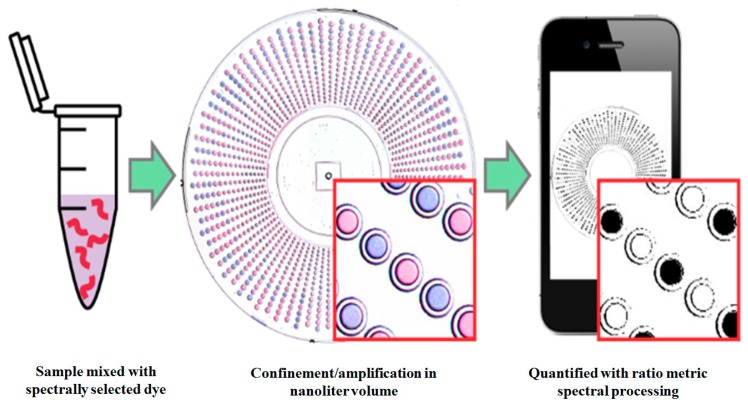
Single-molecule counting after isothermal amplification using an unmodified cell phone camera. (Reproduced with permission from [52]).

As shown in Figure 3, an unmodified cell phone camera is used for single-molecule counting after isothermal amplification. In general, for the detection of RNA or DNA in the samples extracted nucleic acid is mixed with the selected dyes and loaded on a compartmentalized microfluidic device that allows isothermal nucleic acid amplification. After the amplification, positive samples show a blue color, whereas negative samples develop a purple color. The ratio-metric image processing allows the identification of positive and negative samples by white and black color, respectively. The numbers of compartments that show positive results are used for the quantification of initial nucleic acid concentration. This method is validated by using λDNA as a model and HCV RNA as a target. The drawback of this method is that it requires an amplification method such as PCR, loop-mediated isothermal amplification (LAMP).

#### 2.2.1. Nucleic Acid Sequence-Based Amplification (NASBA)

After its first report by Compton J., nucleic acid sequence-based amplification (NASBA) has become popular in the field of molecular diagnostics for RNA targets. Compton J. described NASBA as a primer-dependent technology used for the continuous amplification of nucleic acids in a single mixture at one temperature [53]. NASBA was efficiently used for the identification of bacterial [54] and viral [55] RNA in clinical samples [56]. Even though the RT-PCR has verified to be an excellent tool for detection of viruses, it has several drawbacks including a need of a two-step reaction at varying temperatures during amplification. On the contrary, NASBA is a sensitive, isothermal, transcription-based amplification that system overcame the disadvantages of RT-PCR by using a single-step isothermal RNA-specific amplification process [57]. 

**Figure 4:**
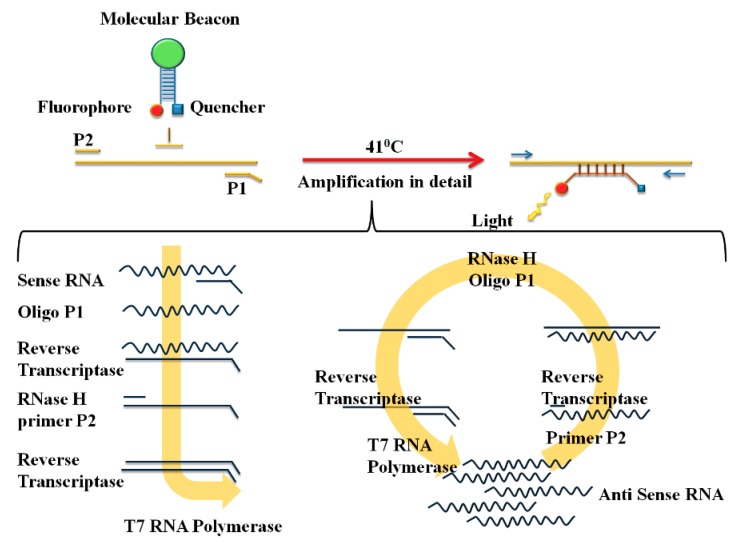
NASBA amplification reaction with the P1 (anti-sense) – P2 (sense) oligonucleotide primer set. The overhang on the P1 encodes the promoter sequence for the T7 RNA polymerase. (Reproduced with permission from [58]).

Figure 4 depicts the amplification and detection of RNA by following NASBA. NASBA is achieved with a primer set consisting of P1 (antisense) and P2 (sense). The P1 encodes the promoter sequence for the T7 RNA polymerase. A real-time detection system for NASBA amplification is generated by using highly specific molecular beacons that hybridize with their amplified target at a relatively low temperature (41 °C) [58].

Guichón et al. reported the NASBA assay for the qualitative detection of HCV in clinical samples. The sensitivity of their assay was 100–150 IU/mL [59]. Even though the sensitivity of this assay is low as compared to previously mentioned RT-PCR, the simplicity of this assay allows direct detection of HCV RNA. The RT-PCR are laborious and generally do not support the standardization required by clinical laboratories. Even though NSBA is a simple method and allows direct detection of HCV RNA, the significant disadvantage of this method is that it does not allow HCV genotyping. Hence, in a recent scenario where it is vital to genotype the HCV for definitive therapy, this method is not suitable for the clinical practice.

#### 2.2.2. Transcription-Mediated Amplification (TMA)

Transcription-mediated amplification (TMA) is an isothermal amplification system that uses two enzymes viz. RNA polymerase and reverse transcriptase for rapid amplification of target RNA/DNA in a single tube [60]. Hence, this method can simultaneously detect multiple pathogenic organisms in a single tube [61]. 

The non-detection of serum HCV RNA by qualitative PCR-based assays is defined as the viral clearance. However, even after achieving apparent viral clearance by the end of treatment, some individuals show recurrence. These individuals may have a low viral load that it is undetected by PCR methods. The TMA based assays are highly sensitive due to their lower detection limits and can detect the levels of viremia below the levels detected by PCR methods. Sarrazin et al. reported that the TMA assay could detect HCV RNA in patients with complete virologic response according to PCR based assay.

Comanor et al. reported that TMA is more sensitive than conventional PCR-based assays for the detection of residual HCV RNA in serum at the end of treatment [62]. They found that the TMA-based assay detected HCV RNA in 34.6% of end of treatment samples that showed apparent viral clearance by PCR [63]. Vinaya et al. reported the evaluation of Versant HCV RNA Qualitative Assay, based on TMA technology, for detection of HCV RNA in the dialysis patients [64]. Hofmann et al. compared Versant HCV RNA Qualitative Assay and RT-PCR for the detection of HCV RNA in formalin-fixed paraffin-embedded liver biopsy samples [65]. In this study, they found the assay based on TMA technology to show higher sensitivity than standard RT-PCR assay.

TMA offers several advantages including the requirement of a small amount of RNA and the assay time, which is less than 4 h. The risk of cross-contamination in the TMA-based assay is very low as it uses a single-tube reaction. Moreover, the cDNA formed during the reaction does not interfere with the assay. The RNA amplicons generated in the TMA reactions are significantly more labile than PCR products. Therefore, the risk of carryover contamination and false positive results is substantially low [66].

#### 2.2.3. Reverse Transcription Loop-Mediated Isothermal Amplification (RT-LAMP)

Loop-mediated isothermal amplification (LAMP) is an isothermal nucleic acid amplification technique. Notomi et al. initially developed this method for the amplification of DNA in a single reaction tube [67]. LAMP amplifies DNA with high specificity, efficiency, and rapidity under isothermal conditions. LAMP, when combined with the reverse transcription step, allows the detection of RNA and the method is called as reverse transcription loop-mediated isothermal amplification (RT-LAMP). Several tests are developed based on this method as it is simple throughout all steps, right from the extraction of nucleic acids to the detection of amplified targets [68,69]. In the LAMP reaction, amplification is done at a fixed temperature with a repetition of two types of elongation reactions occurring at the loop regions.

**Figure 5:**
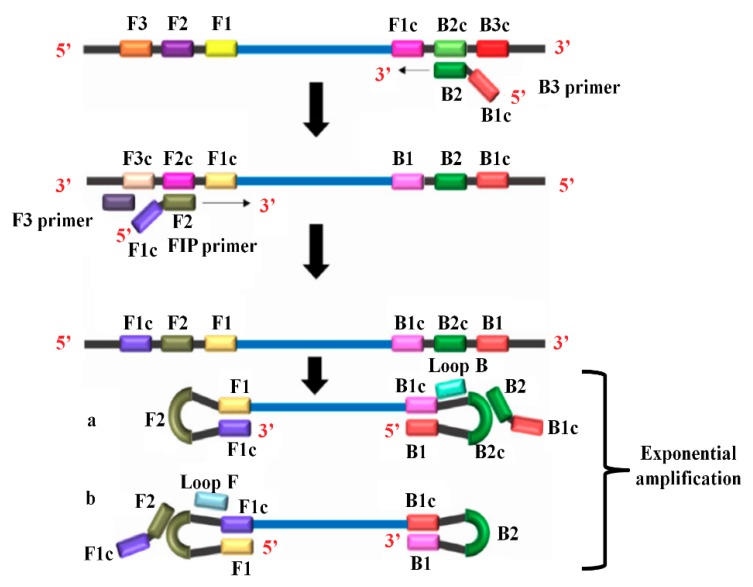
Loop-mediated amplification method. (Reproduced with permission from [70]).

As shown in Figure 5, a LAMP reaction requires a mixture of the dNTPs mix, a fluorescence dye, primers, Bst polymerase, and cDNA template obtained in reverse transcription reaction [70]. LAMP reaction requires four primers that recognize six different regions of the target DNA. Forward inner primer (FIP) consists of F2 and F1c regions at the 3′-end and 5′-end, respectively. Forward outer primer (F3 Primer) comprises F3 region that is complementary to the F3c region of the template DNA. Backward inner primer (BIP) contains a B2 and B1c region at 3′-end and 5′-end, respectively. Backward outer primer (B3 Primer) contains a B3 region that is matching to the B3c region of the template. The amplification reaction starts when the F2 region binds to the F2c region of the template and begins a complementary strand synthesis.

The F3 primer binds to the F3c region of the template, extends, and simultaneously displaces the FIP linked complementary strand. The displaced strand forms a loop at the 5′-end. This loop at the 5′-end acts as a template for BIP. B2 binds to the B2c segment of the template and initiates the DNA synthesis. The reaction leads to the construction of a complementary strand and the opening of the 5′-end loop. Then, B3 hybridizes to B3c region of the target DNA and extends, resulting in the displacement of BIP linked complementary strand, which results in the formation of a dumbbell-shaped DNA. The addition of nucleotides at 3′-end of F1 by Bst DNA polymerase extends the F1 resulting in the opening of the loop at the 5′-end. The dumbbell-shaped DNA is converted to a stem-loop structure as indicated by a and b in Figure 5. This stem-loop structure aids as an originator for LAMP cycling, which is a second stage of the LAMP reaction. The whole process allows the exponential amplification of the target regions in the template.

The LAMP has a wide range of applications, including point-of-care testing, genetic testing, and rapid testing of food and environmental samples [71]. Kargar et al. developed a LAMP-based assay for the detection of HCV RNA and compared it with the conventional nested-PCR [72]. They reported that the sensitivity of the LAMP-based assay, conducted at 63.5 °C for 30–60 min, was comparable to the nested-PCR assay and both assays showed a LoD of 10 copies/mL. The LAMP assay was found to be superior for rapid amplification, simple operation, and easy detection. Further, Nyan et al. developed a LAMP assay for the detection and identification of HCV genotypes 1–6 with the sensitivity and specificity of 91.5% and s 100%, respectively [73]. Yang et al. further modified the LAMP for the detection of HCV RNA by employing accelerating primer, the assay was conducted at 60 °C for 60 min. The LOD of their method was found to be 84 IU/mL, and this assay did not show any cross-detection [74]. Pin et al. have discussed various issues of LAMP assay in their review article [75].

#### 2.2.4. Rolling Circle Amplification (RCA) Method

Lizardi et al. demonstrated that the rolling-circle amplification (RCA) by using DNA polymerase could replicate circularized oligonucleotide probes with either linear or geometric kinetics under isothermal conditions [76]. RCA is an isothermal enzymatic amplification method in which a short DNA or RNA primer is amplified to form a long single-stranded DNA or RNA using a circular DNA template and particular DNA or RNA polymerases. 

**Figure 6:**
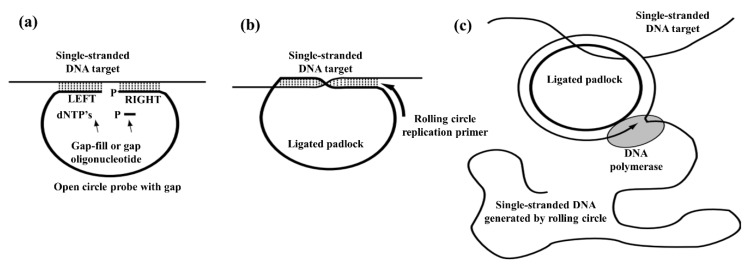
Single-stranded rolling-circle amplification method. (**a**) Binding of a circularizable probe with a small gap to the single-stranded DNA target; (**b**) ligated (padlock) probe, and binding of complementary; (**c**) rolling-circle amplification of a padlock probe by DNA polymerase. (Reproduced with permission from [76]).

As shown in Figure 6, a circularizable probe with a small gap is allowed to bind with the single-stranded target DNA. The gap in the probe is then filled by binding and ligation of small oligonucleotide or by DNA polymerase addition of dNTP’s with simultaneous ligation by DNA ligase. After that, the ligated probe and binding of complementary primer to the single-stranded target DNA allows rolling circle amplification catalyzed by a strand displacing DNA polymerase. Conventionally, RCA was used for the development of highly sensitive diagnostic assays for various targets including DNA, RNA, small molecules, proteins, and cells. Besides these applications, RCA has attracted substantial attention in nanotechnology and nano-biotechnology [77].

Ji et al. reported the application of RCA in combination with Surface plasmon resonance technology for the detection of HCV [78]. The LOD of this method was reported to 1 pmol/L which authors claimed to be lower than the LOD of RT-PCR (0.1 nmol/L) in their study. The sensitivity and specificity of this method were for detection of HCV in clinical samples was 90.0% and 84.8%, respectively.

Overall, isothermal amplification methods are simple and rapid, and hence they are highly applicable for the detection of HCV in clinical settings. Each isothermal amplification method is highly dependent on the enzymatic activity and a primer design to circumvent the need for denaturation and annealing at varying temperatures. The use of lower primer annealing temperatures may result in the non-specific binding of primers, indicated by non-specific amplification, which is not ideal for diagnostics. However, such problems can be solved by using highly efficient enzymes that may be engineered to suit the purpose.

## 3. Transducer Technologies

### 3.1. Bioelectric Recognition Assay (BERA)

The Bioelectric recognition assay (BERA) uses a biosensing system that detects the change in electric signals of cultured cells in reaction matrix, to various ligands that can bind to the cell [79]. 

**Figure 7:**
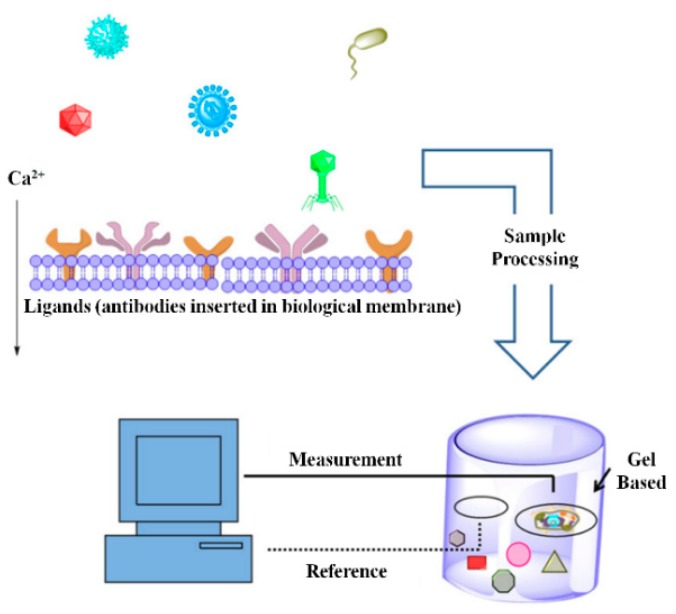
Working principle of Bioelectric Recognition Assay (BERA), a functional principle of an amperometric biosensor. (Reproduced with permission from [80]).

As shown in Figure 7, ligands such as antibodies against a specific pathogen are electro-inserted into a biological membrane (often in living cells) in the first step of BERA development. In the second step, a sample is added to the reaction matrix containing modified biological membranes obtained in the first step. Once the pathogenic cells in samples bind to the antibodies on the biological membrane, calcium flux across the membrane changes due to alteration in membrane structure. The change in calcium flux across the membrane causes change in membrane potential that is detected with high sensitivity [80].

BERA has been used for the detection of viruses by measurement of the change of the electric potential. Kintzios et al. reported a method for the qualitative and quantitative determination of HCV by measuring the change in the electric potential caused by specific interaction with appropriately immobilized viable cells and the HCV [81]. For detection of HCV, they constructed a biosensor under sterile conditions by immobilizing in vitro selected human epithelial cells and liver cells that show a specific response against the HCV. BERA-assays showed high correlation with RT-PCR and ELISA indicated by the correlation coefficient (r^2^) of 0.9201 and 0.8033, respectively. Unfortunately, due to the use of living cells, this method is not suitable for routine clinical investigation of HCV samples.

### 3.2. Piezoelectric Biosensors (PZ) 

**Figure 8:**
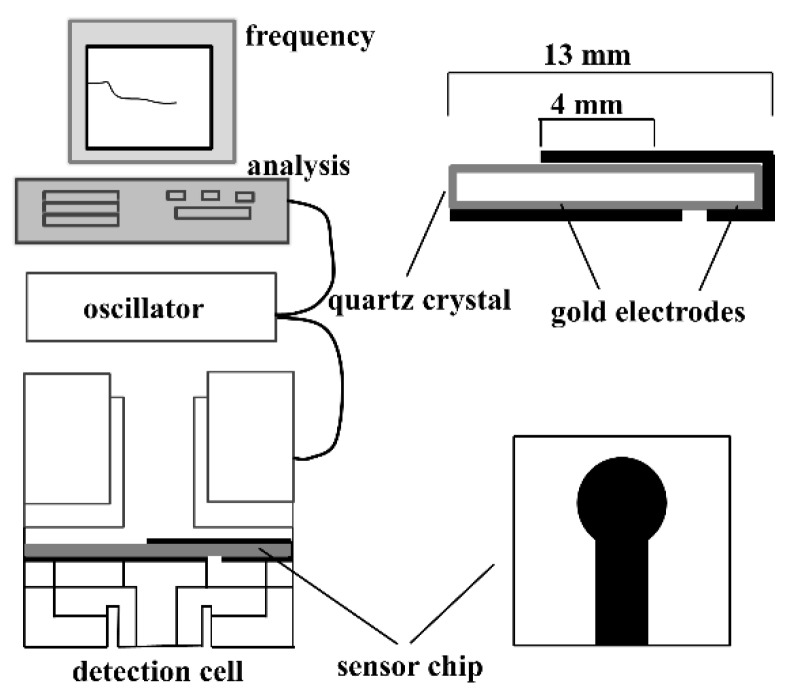
A functional principle of a Piezoelectric biosensor. (Reproduced with permission from [82]).

Piezoelectricity is defined as a direct interaction between the mechanical and electrical systems in a non-centric crystal or similar structures. Piezoelectric (PZ) biosensor is also known as a quartz crystal microbalance (QCM). A PZ biosensor is based on the ability to detect the mass on the surface of the quartz crystal with high sensitivity and specificity after a bio-reaction [82,83]. As shown in Figure 8, the piezoelectric biosensors comprise a transducer that is made of a piezoelectric material (e.g., quartz) and a biosensing material that is coated with the piezoelectric material that vibrates at a natural frequency. The frequency is controlled by an external electrical signal that produces the current. When the target analyte interacts with the sensing material, a shift in frequency results in changes in the current reading that is then correlated with the amount of the analyte.

Skládal et al. reported the piezoelectric quartz crystal resonators decorated with oligonucleotide probes for the detection of HCV in serum samples [84]. They used gold electrodes modified with the self-assembled monolayer (SAM) of cystamine. The glutaraldehyde-based activation of cystamine SAM followed by attachment of streptavidin allows the immobilization of biotinylated DNA probes complementary to the cDNA of HCV. A cDNA generated in RT-PCR can be directly monitored on the biosensor for the detection of HCV in clinical samples within 10 min. The primary advantage of this method is that the same sensing surface is suitable for repeated use. However, the LOD or sensitivity and specificity were not reported for this method.

### 3.3. Amperometric Biosensor 

**Figure 9:**
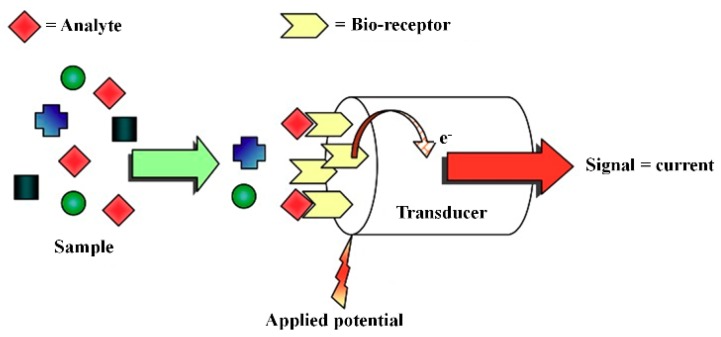
A functional principle of an amperometric biosensor. (Reproduced with permission from [85]).

The signal in the amperometric biosensor is produced by the electron exchange between the electrode and the biological system in the bio-receptor layer. As shown in Figure 9, the analyte undergoes a redox reaction and the change in the current in an electrochemical cell is measured. The analyte that is involved in a biochemical reaction changes its oxidation state at one electrode. The observed electron flux is proportional to the amount of the analyte electrochemically transformed at the electrode [85].

**Figure 10:**
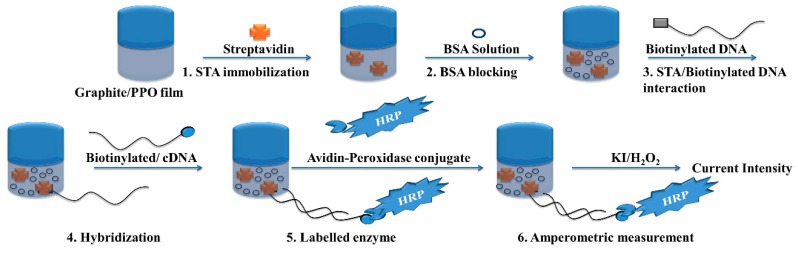
Scheme of HCV detection by amperometric DNA biosensor. (Reproduced with permission from [86]).

Riccardi et al. developed the amperometric biosensor by using streptavidin encapsulated in thin films siloxane-poly(propylene oxide) hybrids prepared by sol-gel method [86]. They deposited these thin films on the graphite electrode surface by dip-coating process. Then biotinylated 18mer oligonucleotide probes were immobilized through streptavidin-biotin binding to generate the amperometric DNA biosensor for the detection and genotyping of HCV as shown in Figure 10. This method allows genotyping of HCV genotypes including genotypes 1, 2A/C, 2B, and 3.

Uliana et al. reported the amperometric biosensor for the detection of HCV using fractional factorial designs [87]. The biomolecules were immobilized on the graphite electrodes pre-modified with siloxane-poly(propylene oxide) hybrid matrix prepared by following the sol-gel method. After optimization of several parameters including the streptavidin concentration at 0.01 mg/mL and 1.0% bovine serum albumin, an incubation time of 30 min, and a 1:1500 dilution of the avidin–peroxidase conjugate, allowed the highly sensitive detection of HCV. Riccardi et al. reported the label-free detection of HCV based on the modified conducting polypyrrole films at microelectrodes and atomic force microscopy tip-integrated electrodes [88]. The LOD of this method was 1.82 × 10^−21^ mol/L and allowed the genotyping of HCV genotypes 2a/c, 2b, and 3 and without any unspecific binding.

### 3.4. Nanotechnology

Nanotechnology convergence of engineering and technology that is conducted at the nanoscale. In particular, nano-biotechnologies are highly applicable in clinical practice for their potential use in the diagnosis of infectious diseases. Nanotechnology includes nanosensors, nanoparticles, protein arrays, nanoarrays. Functional nanoparticles modified with biological molecules have been extensively used in the molecular diagnostics [89,90,91]. 

#### 3.4.1. Gold Nanoparticle (GNP)

Gold nanoparticles (GNP) are known for their unique optical property that produces intense color upon aggregation [92]. GNPs are extensively used in colorimetric assays for the detection of nucleic acids [93,94]. There are several reports on the ways to modify GNPs. They can be modified by using organic cross-linkers [95,96] or by using single-stranded thiol-modified probe [97] for the direct detection of nucleic acids. GNPs were used in the detection of hepatitis viruses including HBV and HCV [98].

**Figure 11:**
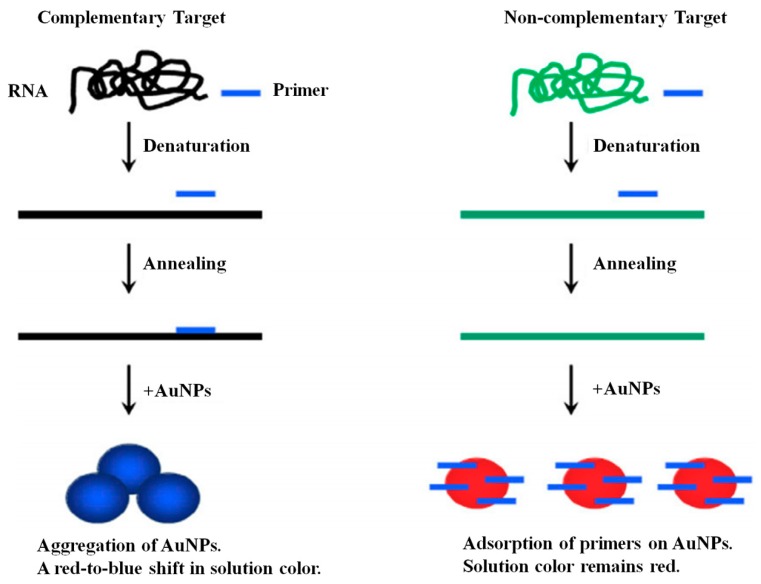
The colorimetric detection of full-length HCV RNA on unmodified GNPs. (Reproduced with permission from [99]).

As shown in Figure 11, Shawky, et al. described a phenomenon that the presence of primers in the solution prevents the aggregation of the GNPs and the solution remains red [99]. However, consumption of primers in the PCR reaction of complementary RNA results in decreased concentration of RNA inducing the aggregation of GNPs and the solution color change from red-to-blue, a positive result. They found that the color of the solution changes from red to blue within 1 min in HCV positive samples. This assay showed a sensitivity and specificity of 92% and 88.9%, respectively, with LOD of copies/reaction.

**Figure 12:**
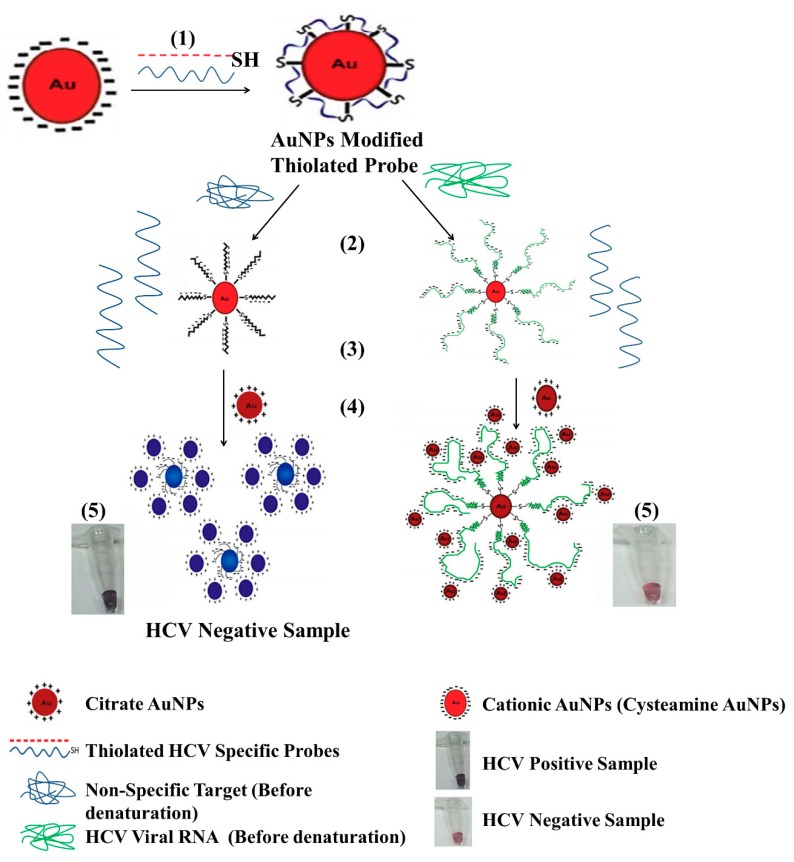
Schematic representation of a principle used in gold nanoparticle (GNP)-based detection of HCV. (Reproduced with permission from [100]).

In another study by Shawky et al. in which they used cysteamine treated GNP’s bearing positive and decorated with HCV RNA specific probe induce the aggregation of citrate-capped GNPs as shown in Figure 12 [100]. This approach allows direct quantification of HCV RNA in clinical samples. The LOD for this method was found to be 4.57 IU/µL that allowed the detection of HCV RNA in clinical samples with a sensitivity and specificity of 93.3% and 100%, respectively. These values were found in agreement with the results of RT-PCR that showed a 96.8% sensitivity and 100% specificity. Even though these methods show excellent sensitivity and specificity for the detection of HCV in clinical specimens, they are not applicable in the current scenario where the genotyping of HCV is of critical importance for definitive therapy.

**Figure 13:**
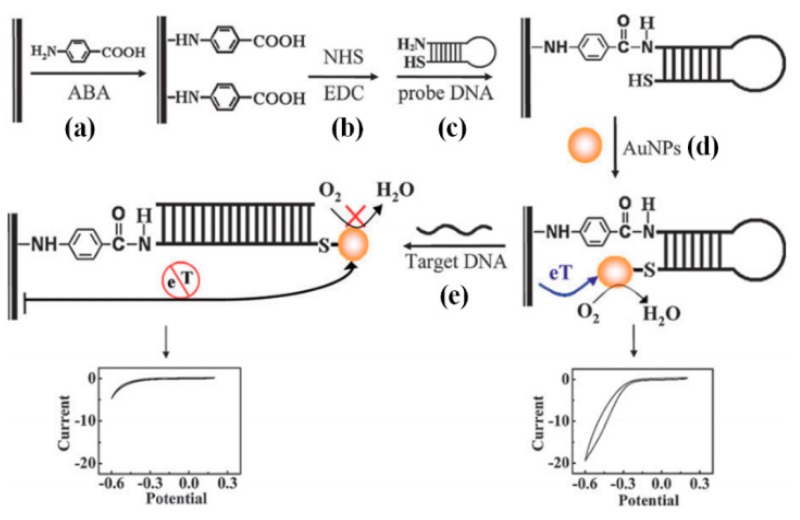
A scheme for the detection of HCV by using GNPs labeled conformation-switched hairpin DNA probe and electrocatalytic signal amplification (Reproduced with permission from [101]).

As shown in Figure 13, Li et al. reported an assay for the detection of HCV by using GNPs labeled conformation-switched hairpin DNA probe and electrocatalytic signal amplification [101]. The GNPs labeled hairpin DNA probe is complementary to the HCV DNA obtained by the RT-PCR. In the absence of target DNA, GNPs remain closed to the electrode surface and allow the electron transfer detected by cyclic voltammetry. However, in the presence of the target DNA, the complementary DNA hybridization destabilizes the hairpin structure that steers GNPs away from the electrode surface resulting in decrease or disappearance of the current. This assay is highly applicable for the detection of HCV in clinical samples. However, for genotyping of HCV, this assay requires tremendous modifications.

#### 3.4.2. Carbon Nanotube and Quantum Dot 

**Figure 14:**
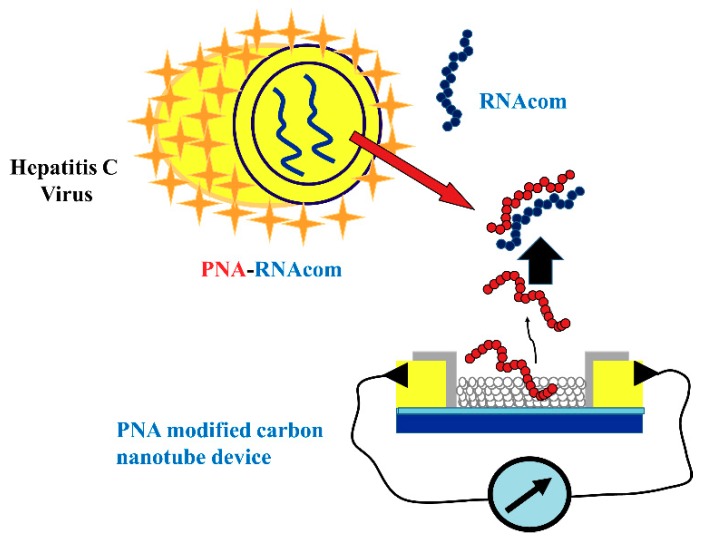
A scheme of peptide nucleic acid (PNA) functionalized single-walled carbon nanotube (SWCNT) field effect transistor device for the detection of HCV (Reproduced with permission from [102]).

Carbon nanotubes (CNT) are divided into two classes including single-walled carbon nanotube (SWCNT) and multi-walled carbon nanotube (MWCNT). There are several reports on the use of CNTs the development of HCV detection assays. As shown in Figure 14, Dastagir et al. reported an SWCNT-based device functionalized with peptide nucleic acid (PNA) that can detect HCV down to the pM range [102]. This method does not allow HCV genotyping.

**Figure 15:**
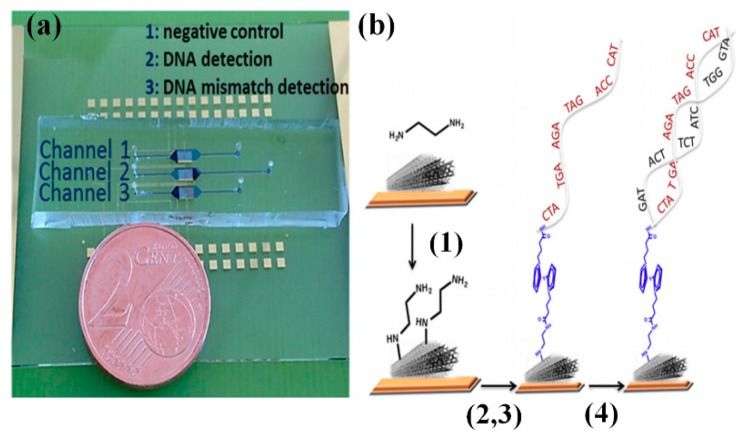
A scheme showing on-chip specific detection of HCV and Mycobacterium tuberculosis (Reproduced from [103]).

Zribi et al. reported a microfluidic-multiplexed platform based on the MWCNT electrochemical sensors for the direct detection of pathogenic viral DNA from Hepatitis C and genomic DNA from Mycobacterium tuberculosis in clinical isolates [103]. As shown in Figure 15, the PDMS/glass point-of-care device contains three channels including (1) a channel for negative control, (2) a channel for DNA detection, and (3) a channel for mismatch DNA detection. Figure 15b indicates the stepwise preparation of device and detection of the target nucleic acid by using immobilized complementary probes. The hybridization of the probe with the target DNA leads to the formation of a double-strand DNA that decreases the charge transfer due to this blocking effect. The primary limitation of this assay is that the HCV genotyping is not possible in its current format.

#### 3.4.3. Graphene

Graphene is widely known for its tunable electrochemical behavior that makes it highly applicable for the development of nanoelectronic devices and highly sensitive electrochemical biosensors. Zribi et al. developed a graphene-based electrochemical biosensing platform for label-free DNA detection from HCV [104]. Even though this assay allows sub-attomolar level HCV detection, it is not applicable for HCV genotyping in its current form and requires several modifications.

**Figure 16:**
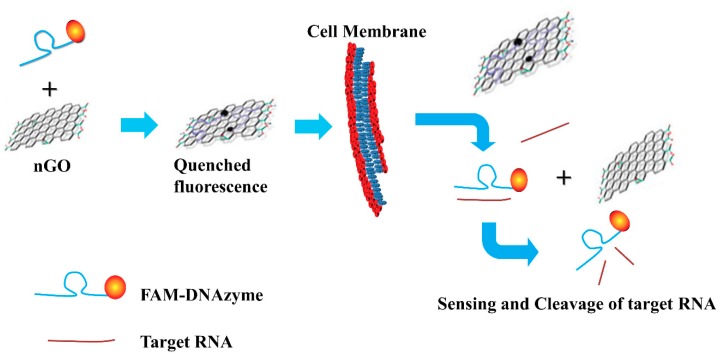
The strategy of detection and knockdown of the target gene based on Dz and nGO (Reproduced with permission from [105]).

Kim et al. developed a method using deoxyribozyme-loaded nano-graphene (Dz/nGO)oxide for simultaneous detection and silencing of the HCV gene in liver cells [105]. As shown in Figure 16, the Dz effectively binds to the surface of nGO resulting in quenching of the fluorescence of a dye conjugated to Dz. In the presence of complementary DNA, Dz detaches from the nGO due to the formation of double helix resulting in the recovery of the fluorescence followed by catalytic cleavage of the target HCV NS3 mRNA sequence. Therefore, the Dz/nGO complex can serve as a sensor to locate and monitor the HCV gene in live mammalian cells.

#### 3.4.4. Branched DNA Signal Amplification Technology (bDNA)

After its development by Chiron in the early 1990s, branched DNA (bDNA) amplification has seen tremendous improvement in its application for the viral load quantification of HCV [106]. A bDNA signal amplification technique is used for the improving the sensitivity of the DNA-based assays by signal amplification. The quantitative hybridization assays based on bDNA signal amplification are extensively used for the detection of various pathogens including HCV [107,108].

**Figure 17:**
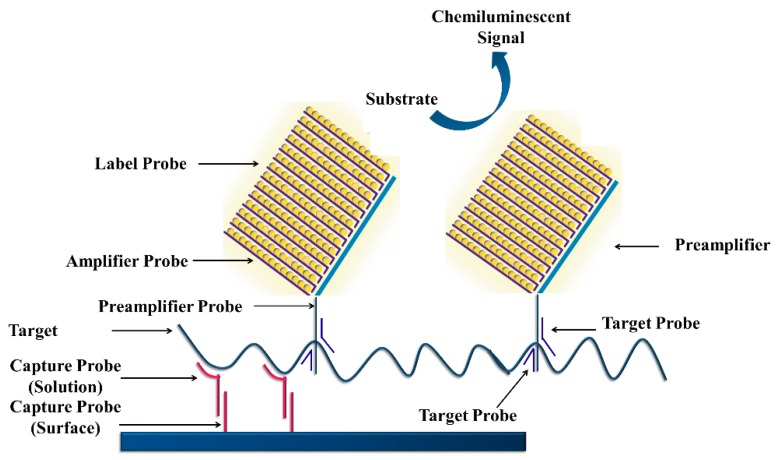
Schematic representation of signal amplification by branched DNA signal amplification technology (Reproduced with permission from [107]).

As shown in Figure 17, a target DNA hybridizes with the capture probes immobilized on the surface [109]. Target probes also hybridize with the pre-amplifier probes; the pre-amplifier probes hybridize to amplifier probes resulting in a branched structure. The alkaline phosphatase-labeled probes are hybridized to this complex in the presence of a chemiluminescent substrate that allows color development.

Sarrazin et al. reported a comparison between RT-PCR assay and bDNA-based assay (Versant Quantitative 3.0) for quantification of HCV RNA in clinical samples and its clinical significance for genotypes 1 to 5 [110]. The RT-PCR assay showed significant under HCV viral load for genotypes 2a/c, 3, 4, and 5. However, the bDNA assay showed reproducible lower quantification of genotypes 1 to 3.

## 4. Lateral Flow Assays

**Figure 18:**
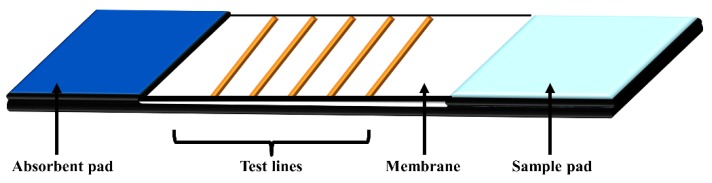
Schematic representation of signal amplification by branched DNA signal amplification technology.

The lateral flow assays (LFA) for point-of-care devices are highly applicable for the qualitative analysis of nucleic acids and quantitative analysis of protein biomarkers. The LFA usually performed over a strip membrane that is assembled on a plastic cartridge. As shown in Figure 18, the LFA is consist of a sample application pad, a membrane (e.g., nitrocellulose, glass fiber) on which the capture probes/antibodies are immobilized, and an adsorption pad. The advantages of LFA include low-cost, simple, rapid, and portability of detection devices.

Stuyver et al. reported the line probe assay (LiPA) that is a reverse-hybridization assay for genotyping of HCV isolates and characterization of new subtypes [111]. LiPA is based on detection of HCV genotypes based on the variations found in the 5′ untranslated regions of the HCV. This assay allows fast and straightforward determination of four HCV genotypes and their subtypes. 

Verbeeck et al. reported a modified version of LiPA 2.0 that uses nucleotide sequence variations in both the 5′ untranslated region and the core region that allows this assay to distinguish between HCV genotype 1 and subtypes c to l of genotype 6 and between subtypes a and b of genotype 1 [112]. A recent report on the performance of the LiPA 2.0 described that this assay has a limitation in identifying HCV genotype 6. Moreover, the LiPA 2.0 cannot distinguish between some HCV genotype 3b samples and HCV genotype 6 samples [113].

Commercial assays including LiPA 2.0, Abbott Realtime HCV Genotype II assay, and Trugene assay are widely used assays for HCV genotyping. However, when the Trugene assay and LiPA 2.0 are compared with sequencing, these assays failed to differentiate HCV subtypes 1a and 1b [114]. The Abbott Realtime HCV Genotype II assay and LiPA 2.0 assay does not have accuracy in the genotyping of HCV 6.

Chantratita et al. reported a 6 HCV genotyping 9G test for the HCV genotyping [115]. The process of 6 HCV Genotyping 9G test includes viral RNA isolation, cDNA synthesis, PCR amplification, and detection of PCR amplicons. The primers used in this test amplify the 5′UTR region of HCV for genotyping of six genotypes.

**Figure 19:**
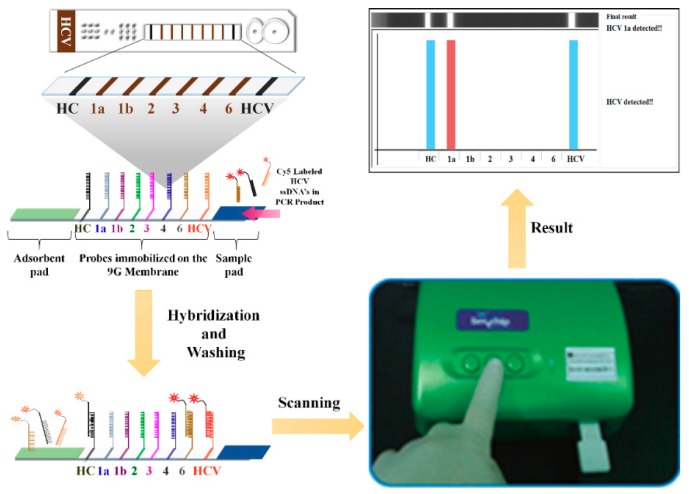
Schematic representation of the working principle and the experimental protocol of 6 HCV Genotyping 9G Test (Reproduced with permission from [115]).

As shown in Figure 19, the HC (hybridization control) probe, HCV (probe specific for the detection of HCV) probe, and six other probes specific to the HCV genotypes 1a, 1b, 2, 3, 4, and 6, are immobilized on the glass fiber membranes according to a reported method [116]. The capture probes selected according to the generalized probe selection method [117] allow highly specific detection of six HCV genotypes. The 6 HCV Genotyping 9G test genotypes six HCV types in 1 PCR in 30 min after PCR amplification [118]. The LOD for the detection and genotyping of HCV 1a, 1b, 2, 3, 4, 6a, 6f, 6i, and 6n genotypes, was reported to be copies/test (38 copies/mL). Therefore, the LOD of the 6 HCV Genotyping 9G test is much lower than the reported for the commercial assays (4810 IU/mL) [119]. 

Warkad et al. reported the performed 6 HCV genotyping test in comparison with the LiPA 2.0 assay and sequencing for HCV genotyping in 280 plasma samples [120]. The sensitivity, specificity, PPV, and NPV of 6 HCV Genotyping 9G test were 99.5, 98.8, 99.5, and 98.8%, respectively. Therefore, the 6 HCV Genotyping 9G test can provide critical information to physicians and assist in the effective hepatitis C treatment.

Table 2 summarizes the LOD, sensitivity, specificity, advantages, and disadvantages of the technologies discussed in this article.

## 5. Conclusions

There has been tremendous research in the last three decades on technologies for the detection and discrimination of HCV. However, a large number of undiagnosed HCV-infected patients poses challenges on the control and treatment of hepatitis C. The current methods for the detection of HCV and quantification of viral load are not sensitive enough to detect viral clearance by the end of treatment as some individuals show recurrence. Hence, there are chances to improve the current technologies that can achieve the limit of detection lower than the current levels to identify patients that show viral clearance by the end of treatment. The critical aspect in the successful treatment of hepatitis C is the correct choice of drug based on the HCV genotype. Hence, identification of HCV genotype in clinical specimens is of supreme importance in achieving viral clearance. The lateral flow assays show higher sensitivity and specificity for HCV genotyping than the conventional methods. Hence, these assays are critical in reducing the global HCV burden. Simplification of the current assays with significant improvement in the sensitivity and specificity will in turn advance the hepatitis C global healthcare practices.

## Figures and Tables

**Table 1:** Comparison of TaqMan^®^ based assay and SYBR^®^-Green based assay.

Parameter	TaqMan^®^-Based HCV detection assay	SYBR^®^-Green Based HCV Detection Assay
Chemistry	Fluorogenic probes are used for detection of specific PCR products	PCR product is detected by SYBR Green I dye that binds to double-stranded DNA with high specificity
Advantages	Low signal to background ratio reduces the number of false positive results. Multiplex detection in a single tube reaction is possible by using distinguishable reporter dyes No post-PCR processing Detects specific amplification products only	- Monitoring the amplification of any double-stranded DNA sequence is possible - No need of probes reduces assay setup and running costs - High sensitivity in the detection of PCR products as multiple dye molecules can bind to single amplicon
Disadvantages	Requires use of multiple probes for target specificity in multiplex assay	Detects any double-stranded DNA, including non-specific reaction products

**Table 2:** Comparison of different methods used for the detection and genotyping of HCV.

Technology	LOD	% Sens.	% Spec.	Advantages	Disadvantages	Ref.
**RT-PCR**	125–500 IU/mL	98.0	100.0	Exponential amplification of template RNA	Inaccurate quantification due to numerous sources of variation	[38,121]
**Real-Time PCR**	28–5000 copies/mL	99.1	100.0	Qualitative and quantitative analysis	Requires expensive equipment and reagents	[46,49,50]
**NASBA**	500 copies/mL	98.0	100.0	Requires single-step isothermal RNA-specific amplification process	Does not allow HCV genotyping, 120–250 nucleotides sequence >250 or <120 amplifies less efficiently	[57,59,122,123]
**TMA**	50 copies/mL	100.0	99.5	Single tube reaction Low-risk of cross contamination	-	[63,66]
**RT-LAMP**	10 copies/mL	91.5	100.0	Rapid amplification Simple operation Easy detection	Effective only when the template is pure	[72,73,74,124]
**RCA**	0.1 nmol/L	90.0	84.8	Aptamer/DNAzyme can replicate hundreds of times in a short time. Improvement of LOD is possible if combined with other biosensors.	Extremely complicated and it is not capable of amplifying a satisfactory length of nucleic acids	[78,125,126]
**BERA**	-	-	-	High operational speed	Chances of cross-contamination are high (require separate space)	[79]
**PZ**	-	-	-	Can be modified in different assay formats including direct detection, label free detection etc.	High temperature sensitivity	[127,128,129]
**AB**	1.82 × 10^−21^ mol/L	-	-	High selectivity for substrates	Used biosensors exhibit limited stability.	[88,130]
**GNP**	4.57 IU/µL	93.3	100	Assay is highly applicable for the detection of HCV	Not applicable for genotyping in its current form	[100]
**CNT**	0.5 pM	-	-	Detection of HCV to picomolar range	HCV genotyping is not possible	[102]
**Graphene**	0.1 nmol/L	-	-	Can serve as a sensor to locate and monitor the HCV gene in live mammalian cells	Not applicable for HCV genotyping in its current form	[104,131]
**bDNA**	3.2 × 10^3^ copies/mL	-	98.2	Low non-specific interactions. Multiple sample detections at ones	High LOD	[107]
**LFA**	38 copies/mL	99.5	98.8	Low-cost, simple, rapid, and portable	Quantification is not possible	[115]

Sens., Sensitivity; Spec., Specificity; RT-PCR, Reverse transcription polymerase chain reaction; NASBA, Nucleic acid sequence-based amplification; TMA, Transcription-mediated amplification; RT-LAMP, Reverse transcription loop-mediated isothermal amplification; RCA, Rolling circle amplification; BERA, Bioelectric recognition assay; PZ, Piezoelectric biosensors; AB, Amperometric biosensor; GNP, Gold nanoparticle; CNT, Carbon nano-tube; bDNA, Branched DNA signal amplification technology; LFA, lateral flow assay; LOD, limit of detection.
